# The fecal microbiota as a biomarker for disease activity in Crohn’s disease

**DOI:** 10.1038/srep35216

**Published:** 2016-10-13

**Authors:** Danyta. I. Tedjo, Agnieszka Smolinska, Paul H. Savelkoul, Ad A. Masclee, Frederik J. van Schooten, Marieke J. Pierik, John Penders, Daisy M. A. E. Jonkers

**Affiliations:** 1 School of Nutrition and Translational Research in Metabolism (NUTRIM), Division Gastroenterology-Hepatology, Maastricht University Medical Center+, Maastricht, The Netherlands; 2 School of Nutrition and Translational Research in Metabolism (NUTRIM), Department of Medical Microbiology, Maastricht University Medical Center+, Maastricht, The Netherlands; 3 School of Nutrition and Translational Research in Metabolism (NUTRIM), Department of Pharmacology & Toxicology, Maastricht University Medical Center+, Maastricht, The Netherlands

## Abstract

Monitoring mucosal inflammation is crucial to prevent complications and disease progression in Crohn’s disease (CD). Endoscopy is the current standard, but is invasive. Clinical activity scores and non-invasive biochemical markers do not correlate well with mucosal inflammation. Microbial perturbations have been associated with disease activity in CD. Therefore, we aimed to investigate its potential use to differentiate CD patients in remission from those with an exacerbation. From 71 CD patients repeated fecal samples were collected, resulting in 97 active disease and 97 remission samples based on a combination of biochemical and clinical parameters. The microbiota composition was assessed by pyrosequencing of the 16S rRNA V1-V3 region. Random Forest analysis was used to find the most discriminatory panel of operational taxonomic units (OTUs) between active and remission samples. An independent internal validation set was used to validate the model. A combination of 50 OTUs was able to correctly predict 73% of remission and 79% of active samples with an AUC of 0.82 (sensitivity: 0.79, specificity: 0.73). This study demonstrates that fecal microbial profiles can be used to differentiate between active and remission CD and underline the potential of the fecal microbiota as a non-invasive tool to monitor disease activity in CD.

In the past decades, the incidence of Inflammatory bowel diseases (IBD), comprising Crohn’s disease (CD) and ulcerative colitis (UC), has been increasing in industrialized countries in Europe and North America. Currently, a rise has also been reported in Asian countries, in line with westernization^1^,^2^. UC is characterized by continuous mucosal inflammation in the colon, while CD can affect any part of the gastrointestinal tract and can be transmural and discontinuous. Both UC and CD are associated with periods of active inflammation with symptoms such as abdominal pain and (bloody) diarrhea, alternated with periods of remission^3^. Treatment is merely symptom-based and focuses on inducing or maintaining remission. However, current treatment modalities are associated with mild to severe side effects and limited long-term efficacy^4^,^5^. Thereby, IBD has a significant impact on the patient’s quality of life and accounts for substantial costs to the health care system, especially during exacerbations^6^.

Monitoring mucosal inflammation is crucial to limit disease progression and complications. Endoscopy is the current standard, but is an expensive and invasive procedure with risk of complications^7^.

Clinical activity scores, such as the Harvey-Bradshaw index (HBI) for CD and the simple clinical colitis activity index (SCCAI) for UC, are often used in clinical practice and therapeutic intervention trials, but do not correlate well with mucosal inflammation^8^. In daily clinical practice, inflammatory markers such as C-reactive protein (CRP) and fecal calprotectin (FC) are often used to evaluate disease activity. CRP, however, is not specific for intestinal inflammation^9^,^10^. FC correlates well with endoscopic scores in UC, but its’ diagnostic accuracy is less for CD due to a limited sensitivity for the proximal colon and small bowel^9^,^10^. Therefore, new non-invasive markers for active disease are needed, especially for patients with CD.

Current biochemical markers used to monitor disease activity, are often non-specific and not associated with possible pathophysiological mechanisms. Nowadays, it is generally accepted that the microbiota plays an important role in the development and disease progression of IBD^11^,^12^. According to previous studies the microbiota composition of CD patients is characterized by a decrease of fecal and mucosal microbial diversity and a change in the relative abundance of specific bacterial taxa (*e.g*. reduction of *Faecalibacterium prausnitzii*) compared to the microbiota of healthy individuals^13^,^14^,^15^. Furthermore, also clear differences have been reported in active versus quiescent disease, although results between studies are inconsistent, most likely due to methodological differences^16^,^17^,^18^,^19^,^20^,^21^,^22^,^23^,^24^,^25^. A study by Swidsinski *et al*. showed that concentrations of mucosal associated bacteria increased with disease severity^26^. Moreover, antibiotics are able to induce remission in active CD patients and are effective against anal lesions and in the prevention of post-operative recurrence CD^27^. These studies suggest that the microbiota plays an important role in inducing exacerbations.

Possible differences in the microbiota composition related to disease activity may result in markers for disease monitoring. So far, specific bacterial taxa clearly associated with disease activity have not been identified yet. Investigating the microbial community structure (i.e. combinations of OTUs) rather than specific microbial taxa might be more effective in investigating the role of the intestinal microbiota in IBD, as previous studies have demonstrated^28^,^29^.

Papa *et al*. was able to distinguish paediatric IBD patients in remission and during an exacerbation as defined by clinical indices with an AUC of 0.72 based on the fecal microbiota composition^28^. However, it was previously shown that CD and UC patients have a different microbiota structure and by collating CD and UC patients together, the classification might not be optimal^30^,^31^. A second study in paediatric CD patients, was able to predict an exacerbation within six months after diagnosis based on the fecal microbiota with an accuracy of 67%^29^. Studies using the fecal microbiota to predict disease activity in adults are lacking. Therefore, the aim of the present study was to investigate the potential use of microbiota profiling to accurately differentiate between Crohn’s disease patients in remission from those with an exacerbation.

## Material and Methods

### Study population

A total of 194 fecal samples (97 remission, 97 active) from 71 CD patients were included in this study. IBD was diagnosed based on clinical and endoscopic or radiological findings conform the ECCO guidelines^32^. These patients were part of a prospective follow-up cohort of IBD outpatients of the population-based IBDSL cohort^33^,^34^. Clinical data, blood and feces were collected at each visit to the outpatient clinic and during an exacerbation. Fecal samples were collected by the patients at home and brought to the hospital within 24 hours after defecation. Upon arrival, part of the sample was sent to the laboratory of Clinical Chemistry for routine analysis of CRP and FC. The remaining part was aliquoted and frozen directly at −80 °C for microbiota analyses. For the purpose of the present study, fecal samples collected within 1 month after a course of antibiotics were excluded.

Baseline demographics, data on disease phenotype, medication use and clinical activity scores were retrieved using the standardized computer registration of the IBDSL cohort^33^. Disease activity was defined by the Harvey Bradshaw index (HBI) in combination with serum CRP or FC^34^. Active disease was defined by a FC > 250 μg/g^35^. Remission was defined by a HBI≤4 in combination with both serum CRP < 5 mg/l and FC < 100 μg/g.

### Ethical statement

The patients included in the present study gave written informed consent prior to participation. The study has been approved by the Medical Ethics Committee of Maastricht University Medical Center+ and is executed according to the revised declaration of Helsinki (59^th^ general assembly of WMA, Seoul, South Korea, Oct. 2008). The study has been registered in the Central Committee on Research Involving Human Subjects (CCMO) registry under file number NL24572.018.08.

### DNA isolation of fecal samples

Frozen aliquots of fecal samples were cut on ice to prevent thawing of the fecal samples and approximately 200 mg was added to vials containing PSP lysis buffer (Stratec Molecular, Berlin, Germany), 0.5 g of 0.1 mm zirconia/silica beads and 4 glass beads of 3.0–3.5 mm (BioSpec, Bartlesville, USA). The fecal samples were homogenized in a MagNALyser instrument (Roche, Basel, Switzerland) in three cycles of 1 min at a speed of 5500 rpm. Samples were kept on ice for one minute in between cycles. DNA isolation was continued using the PSP Spin Stool Kit (Stratec Molecular, Berlin, Germany) according to the manufacturers’ instructions. DNA was finally eluted in 200 μl TE-buffer. Negative control samples (PCR grade water) were included in each batch of samples for DNA-isolation, and handled in exactly the same way as the fecal samples, in order to rule out contamination during the isolation procedure.

### 454 pyrosequencing

Amplification of the V1-V3 16S rRNA amplicons was performed using forward primers consisting of a 9:1 ratio mixture of 8F and 8F-Bif, respectively, and reverse primer 534R as described previously^24^. The PCR reaction was performed using 1x FastStart High Fidelity Reaction Buffer, 1.8 mM MgCl_2_, 1 mM dNTP solution, 5 U FastStart High Fidelity Blend Polymerase (Roche, Indianapolis, USA), 0.2 μM forward primer, 0.2 μM reverse primer and 1 μl of template DNA (15–50 ng/uL) under the following conditions: denaturation at 94 °C for 3 minutes, followed by 25 cycles of denaturation at 94 °C for 30 seconds, annealing at 51 °C for 45 seconds and extension at 72 °C for 5 minutes. The final elongation step was at 72 °C for 10 minutes. Negative controls were included in each PCR run by replacing 1 uL DNA by PCR grade water.

The amplicons were purified using AMPure XP purification according to the manufacturer’s instructions and eluted in 25 μl TE. Amplicon concentrations were determined by Quant-IT Pico Green dsDNA reagent kit (Invitrogen, New York, USA) using the Victor3 Multilabel Counter (Perkin Elmer, Waltham, USA). Thereafter, amplicons were mixed in equimolar concentrations to establish an equal representation of each sample for the emulsion PCR (emPCR). After emPCR (Titanium emPCR Kit (Lib-L)), pyrosequencing was performed according to the manufacturer’s instructions (Roche, Brandford, USA).

### Data presentation and statistical analyses

Baseline demographics and disease phenotype at time of inclusion of the CD patients with active disease versus remission are presented as median and range for continuous variables and numbers and percentages for categorical variables.

The V1-V3 16S rDNA bacterial sequences that were used in this paper have been submitted to the European Nucleotide Archive (ENA) under accession PRJEB11845.

The raw pyrosequencing reads were passed through quality filters using Mothur version 1.32.1 to reduce error rates [1]. Sequences with perfect proximal primer fidelity, a minimum average quality score of 25 over a window size of 50 nucleotides, a read length between 200 and 590, a maximum of one ambiguous base call and a maximum homopolymer length of 6, were retained for further analyses. Sequences were de-multiplexed and clustered by UCLUST algorithm into operational taxonomic units (OTUs) based on 97% similarity against the Greengenes reference set version August 2013 in Qiime 1.8. [2]. Default parameters for UCLUST were applied apart for the following parameters: maxrejects = 100 and stepwords = 16. Sequences that did not cluster to reference sequences were discarded to reduce the influence of sequencing errors.

To control for variation in sequencing effort the OTU-table was subsequently rarefied to 4,930 sequences/sample.

Random Forest (RF) analysis was used to find the most discriminatory OTUs between CD patients with active disease versus remission. As it is unlikely that an OTU present in a minority of samples will have group-related importance, OTUs were only included in the statistical analysis if they were detected in at least 20% of the samples in one of the groups. Prior to actual RF analyses, the microbiome data were transformed via an inverse hyperbolic sine transformation and then mean centered per individual patient^36^. The first step accounts for skewness and can deal with sparse microbiome data. The mean centering per individual diminishes the influence of inter-individual variation.

In the current study, two different RF models were built. The first RF model (with 700 trees), based on 90 different randomly selected subsets, aimed to find the most discriminatory OTUs between active CD and CD in remission. The second RF model was performed to demonstrate the contribution of the most discriminatory OTUs in differentiating active and inactive CD and to test the classification performance of the model in the validation set. The second RF model (with 700 trees) was based on 300 randomly selected subsets. For both RF models, each subset contained all samples from the same individual either in the training set, consisting of 80% of all samples, or in the validation set (the remaining 20%). Thereby, the RF classification model was never trained on part of the measurements of one subject and tested on the remaining measurements of that subject.

The final classification of each sample was determined by a majority of votes (>50%) from 300 RF classification models. The final performance of the RF classification model is demonstrated by the receiver operating characteristic (ROC) curve.

After tree construction, RF computes the proximities, which indicate the similarity between samples. The proximities obtained from the second RF analyses, were used to visualize the differences between the two groups (active or remission) by Principal Component Analysis (PCA).

A canonical correlation analysis (CCA) was performed to check whether the selected OTUs correlated with FC concentrations as indicator of intestinal inflammation.

The directions of bacteria changes in CD patients in active and remission was investigated via boxplot analysis, where the distributional characteristics and the count of bacteria can be shown^37^.

The potential confounding effect of medication use at the time of sampling (*i.e*. use of biologicals (anti-TNF), mesalazines and thiopurines), disease location (ileal (L1), colonic (L2), ileocolonic (L3)) according to the Montreal classification), colectomies and age at time of sample collection, on each of the individual 50 OTUs associated with disease activity was tested using the Friedman test with post-hoc correction for multiple testing. To test whether the set of discriminatory OTUs was statistically influenced by the possible confounding factors (i.e. use of medication, disease location, colectomies and age), we used regularized multivariate analysis of variance (rMANOVA)^38^. For age, the patients were grouped into three classes: patients younger than 30, those in age range 30–50 and patients over ≥50 years of age. A false discovery rate (FDR) cut off value of 0.05 was used to correct for multiple testing.

All analyses were done in Matlab2014a.

## Results

### Study population

A total of 194 fecal samples of 71 Crohn’s disease patients (18–70 years) were included in this study. Baseline characteristics of the 71 patients are presented in Table 1. A single sample was available for 14 patients, whereas for the remaining patients between two to eight fecal samples were collected for the purpose of this study. In total, 97 active and 97 remission samples were available for the analysis. Patient characteristics at time of collection of all 194 samples are given in Table 2. Three patients received a course of antibiotics between 1–3 months prior to collection of one of their remission samples (amoxicillin 5 weeks, daptomycin 8 weeks and ciprofloxacin 12 weeks prior to sample collection, respectively), whereas none of the active disease samples were collected within three months after a course of antibiotics.

### Microbial composition and diversity

A total of 2,617,664 raw sequences were obtained, and after quality filtering and binning 1,616,532 sequences were retained for further analyses with an average of 8,333 sequences per sample (range 4,938–17,8913 sequences/sample). Sequences were clustered into 6,629 OTUs, subsequently singletons were removed and the data were rarefied to 4,930 sequences/sample to control for variations in sequencing efforts.

The fecal microbiota of remission and active samples did not significantly differ with respect to microbial diversity as assessed by Chao1 (median [interquartile range]: 1077.7 [760.6–1280.0] and 1120.2 [823.2–1307.8, resp.] and Shannon indices (7.0 [6.2–7.5] and 6.9 [6.4–7.7], resp.).

With respect to the microbial composition, both remission and active samples were dominated by the phyla Bacteroidetes (relative abundance 52.9% vs. 49.5%, resp.) and Firmicutes (relative abundance 41.0% vs. 42.9% resp.), followed by Proteobacteria (relative abundance 4.6% vs. 5.4%, resp.) and Actinobacteria (relative abundance 0.7 vs. 0.8%, resp., Supplementary Figure 1A). However, the presence of some of the less abundant bacterial phyla differed between the remission and active samples. Fusobacteria could be detected in 31 (32.0%) of the samples collected during active disease, whereas only 6 (6.2%) of the remission samples were positive. In contrast, Verrucomicrobia were more prevalent in remission than in active samples (44.3% vs. 15.5%, resp., Supplementary Figure 1B). Also when only examining the baseline samples of the 71 patients (of whom 35 had active disease at baseline), these differences persisted for both the Fusobacteria (37.1% of active vs. 11.1% of remission samples, p = 0.01) and Verrucomicrobia (17.1% of active vs. 50.0% of remission samples, p = 0.003). The difference in the prevalence of these bacterial phyla was completely driven by the genera *Fusobacterium* and *Akkermansia*, respectively. These genera however did not belong to the dominant microbiota. The microbiota in both remission and active samples was dominated by the genera *Bacteroides, Prevotella* and *Parabacteroides* within the Bacteroidetes phylum en members of the Lachnospiraceae and Ruminococcaceae families within the Firmicutes phylum (Supplementary Figure 1C).

### Random forest analysis

**Figure 1:**
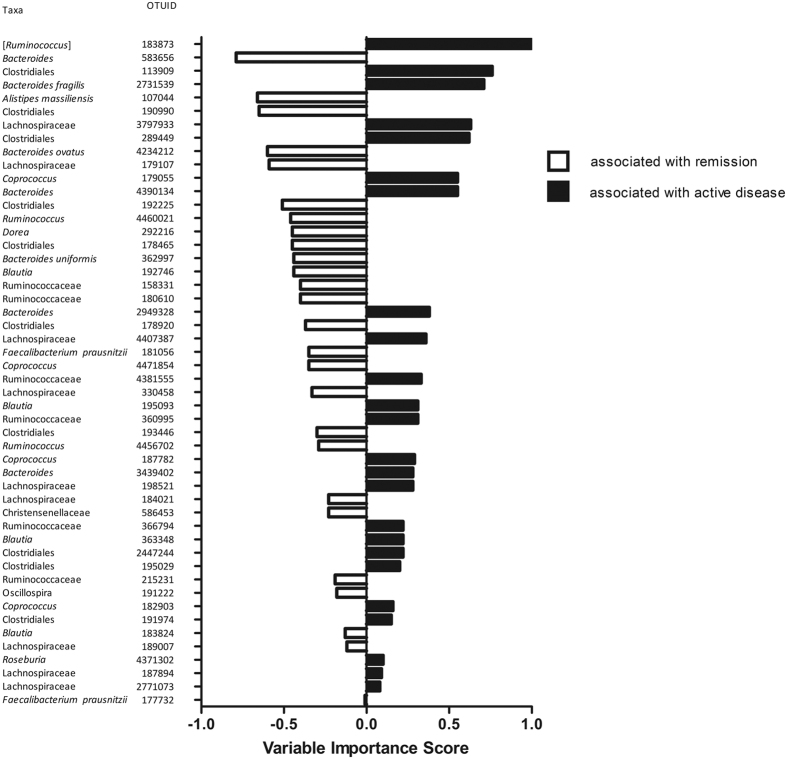
50 most discriminative OTUs, as identified through Random Forest Analysis, to differentiate fecal samples from CD patients during active disease versus remission.

We subsequently performed RF analysis to examine whether we could discriminate samples collected during remission and active disease based upon the microbiota composition. First, we reduced the data by including only those OTUs (n = 1,116) that were present in at least 20% of the remission and/or active samples. Subsequently, a first RF analysis was used for the selection of the most discriminatory OTUs between active and remission samples. The RF-analysis assigned a variable importance score to each OTU, indicating to what extend the OTUs contributed to the model. Based on the variable importance profile, fifty OTUs with the highest variable importance scores were selected (Fig. 1).

**Figure 2:**
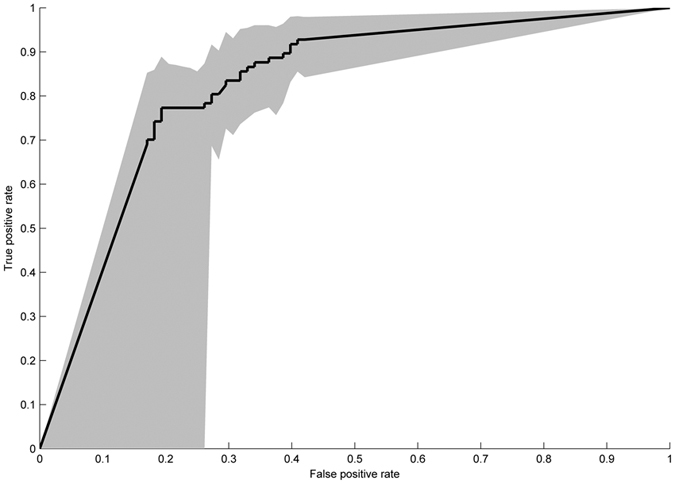
ROC curve for the independent validation set (N = 88 remission and N = 97 active samples) based on the 50 most discriminative OTUs. AUC: 0.82, sensitivity: 0.79, specificity: 0.73.

The performance of the RF classification model based on the most discriminatory OTUs resulted in an area under the ROC curve (AUC) of 0.82 for the validation set, corresponding to a sensitivity of 0.79 and a specificity of 0.73 (Fig. 2). The positive predictive value (PPV) and negative predictive value (NPV) were both 0.76.

**Figure 3:**
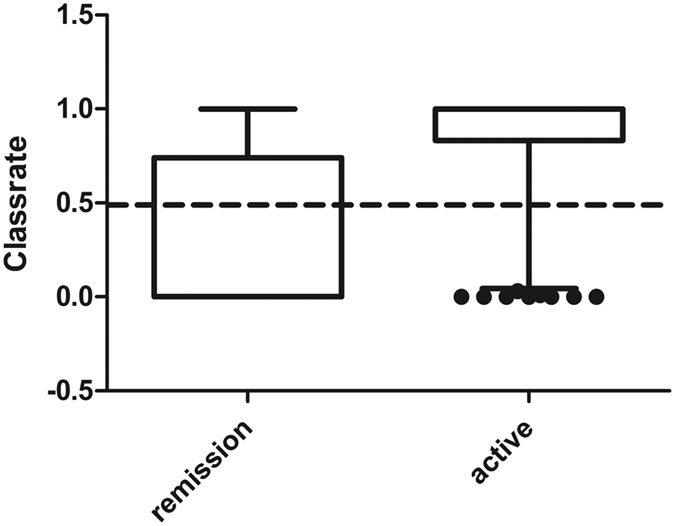
Classification rate of remission (N = 88) and active samples (N = 97) from the independent validation set based on the final RF-model. Classification rates range from 0 to 1. Remission samples with a classification rate <  0.5 were correctly classified as a remission sample. Active samples with an classification rate >0.5 were correctly classified as an active sample.

The prediction rate of each sample ranges from 0 to 1 and is shown in Fig. 3. Samples with a prediction rate of < 0.5 were classified as remission while samples with a classification rate of >0.5 are classified as an active sample. None of the samples had a prediction rate of 0.5.

The most discriminant OTUs with their variable importance scores, colored based on their presence in remission or active samples, are depicted in Fig. 1. OTUs belonging to members of *Lachnospiraceae* and *Ruminococcaceae* were found in both remission and active samples. OTUs classified as *Alistipes massiliensis, Faecalibacterium prausnitzii, Bacteroides ovastus* and *Bacteroides uniformis* were associated with remission samples, whereas other OTUs within the genus *Bacteroides*, including *B. fragilis*, were associated with active samples.

**Figure 4:**
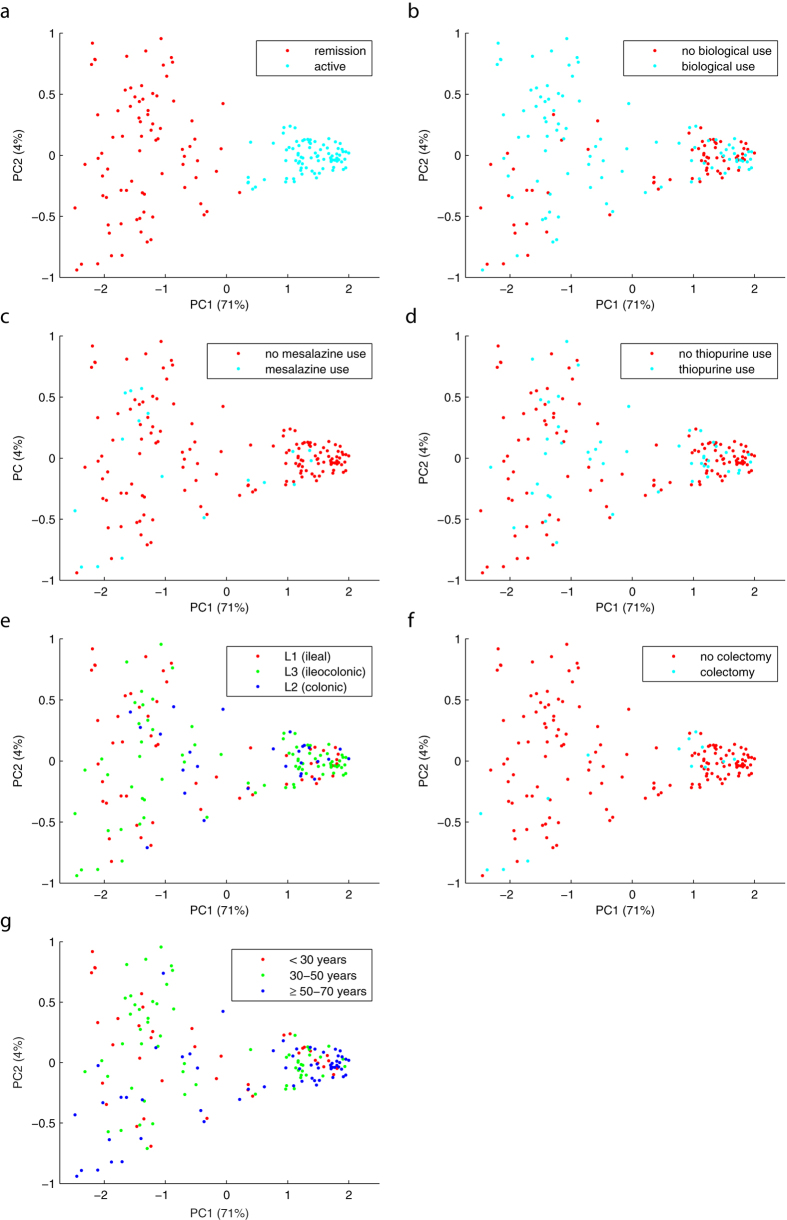
PCA plots based on the proximity matrix from the fecal samples (N = 164) of the training set using the 50 most discriminant OTUs. Samples show a clear separation for active versus remission based on the 50 pre-selected OTUs (**a**), while no separation was observed for use of biologicals (**b**), mesalazines (**c**), thiopurines (**d**), disease location (**e**) colectomy (**f**), and age (**g**).

The principal component analysis (PCA) on the proximities showed a clear separation between active and remission samples (Fig. 4a). Furthermore, the active samples were found to cluster more tightly together than the remission samples, indicating that the inter-sample variation was smaller in the active as compared to the remission samples.

The number of samples of CD patients during remission and active disease that were positive for these discriminative OTUs, as well as the average read numbers of these OTUs per sample, are shown in supplementary Table S1. Some OTUs show clear differences in mean read numbers (e.g. *Bacteroides ovatus* #4234212 and *Bacteroides* #2949328, while for others the differences are not so distinct (e.g. Lanchospiraceae #2771073). This indicates that the entire set of 50 OTUs contributes to the differentiation between active and inactive CD.

Using the Friedman test, no confounding effect due to medication use (i.e. biologicals (p = 0.19), mesalazines (p = 0.54) and thiopurines (p = 0.57)), colectomies (p = 0.55), disease location (p = 0.98) or age (p = 0.45), was observed. The analysis of rMANOVA did not yield significant associations between medication use (biologicals (p = 0.52), mesalazines (p = 0.55) and thiopurines (p = 0.75)), disease localization (p = 0.43), colectomies (p = 0.72) or age (p = 0.72) and the discriminatory set of 50 OTUs. Clustering according to medication use, disease localization, colectomy and age could also not be found in the PCA plots (Fig. 4b–g). CCA analysis showed a strong but non-significant correlation between fecal calprotectin measurements and the 50 most discriminating OTUs (p = 0.16, R = 0.91).

## Discussion

In this study, we demonstrate the potential of fecal microbial profiles as marker for disease activity in patients with CD. Using Random Forest analyses, a combination of 50 bacterial taxa was found to be able to distinguish between active and remission samples with an AUC of 0.82, corresponding to a sensitivity of 0.79 and a specificity of 0.73. Despite the different disease locations and medications used by the study population, the discriminative power of the model was not influenced by these factors, reinforcing that the fecal microbiota has potential as a robust disease activity marker.

A large group of well-characterized CD outpatients from daily clinical practice was included in the present study. Although determination of disease activity by endoscopy is the current standard, this is not feasible in a real-life outpatient follow-up cohort. Therefore, we used a combination of inflammation markers (FC and CRP) and clinical symptoms (HBI) to assess disease activity, which is nowadays well accepted as a surrogate for mucosal inflammation^10^,^35^.

Although previous studies reported an association between specific bacterial taxa and disease severity, others were not able to find such differences^16^,^17^,^19^,^20^,^21^,^22^,^24^,^39^. Most of these studies used univariate analysis methods and compared within and between samples diversity measurements with unsupervised classification methods, which can fail to extract relevant interactions from highly complex data sets. A recent study by Kolho *et al*. found a significant correlation between a combination of 9 bacterial taxa and calprotectin concentrations, while no correlation was found with individual bacterial taxa, highlighting the importance of multivariable analysis of microbiota data^40^.

Supervised learning techniques, suitable to handle highly complex and sparse data sets, have until recently rarely been used in microbiota data analysis^41^. Random forest uses pattern recognition to discriminate between classes and is able to build predictive models such as needed for biomarker discovery. When applying random forest, we found that a combination of 50 bacterial taxa being able to distinguish active from remission samples in adult CD patients with a sensitivity of 0.79 and specificity of 0.73. The performance of our model was even slightly better than comparable analyses in pediatric IBD patients^28^. Our results support the current notion that a combination of bacterial taxa, rather than specific microorganisms, is involved in CD pathogenesis. Our findings were further supported by the PCA plot showing a clear separation between active and remission samples. Interestingly, the microbiota of active samples was found to be more homogenous than the microbiota of remission samples. This indicates a rather individual microbiota composition in CD patients during remission, while during exacerbation patients have a more common microbiota profile. Although previous studies have demonstrated that IBD specific therapeutic interventions, such as mesalazine, antibiotics and thiopurines, can affect the microbiota^24^,^42^,^43^, we found no effect of mesalazine, thiopurines or biological use on the 50 discriminating OTUs as demonstrated by PCA plots. The effect of antibiotics on the 50 discriminating OTUs is unlikely, since none of the patients used antibiotics within a period of 1 month prior to sampling and only three patients, accounting for three fecal samples, used antibiotics between 1–3 months prior the sampling moment. Also no confounding effect of disease location, prior colectomies or age at time of sample collection was found. This supports the potential of this microbial profile as a robust biomarker for active disease. It should however be noted that we cannot exclude any effect of medication use or disease location on the overall microbiota community structure.

The dataset used in our study included multiple measurements for most individuals, which can lead to an overestimation of the results due to the large inter-individual and small intra-individual variation in intestinal microbiota composition^44^. To address this problem, the random forest was performed with a separate training and validation set, in which the model was never trained on part of the samples of one subject while validated on the remaining samples of the same subject^45^,^46^. However, further validation of our microbial biomarker pattern in an independent cohort, using endoscopy as standard, is needed.

The 50 most discriminatory taxa identified in the present study, include both commensal microorganisms as well as opportunistic pathogens, further indicating that merely detecting presence or absence of specific taxa is not sufficient. The 50 OTUs include several bacterial taxa that have previously been associated with disease activity in CD patients, including Lachnospiraceae, *Ruminococcus*, *Roseburia, Blautia, F. prausnitzii* and *B. fragilis*^18^,^20^,^24^,^28^,^47^. However, none of the OTUs belonged to the phyla Verrucomicrobia or Fusobacteria, phyla that were shown to differ in abundance between active and remission samples in our study. This can be explained by the low prevalence of the individual OTUs within these phyla, resulting in the exclusion of these OTUs during the data reduction step prior to the RF analysis.

We found *F. prausnitzii* to be associated with remission. Previous studies have demonstrated that a reduction of *F. prausnitzii* is associated with IBD^18^,^48^,^49^,^50^,^51^,^52^,^53^,^54^,^55^. Furthermore, multiple studies reported reduced *F. prausnitzii* levels in CD patients during active disease in feces and intestinal tissues, suggesting an association between *F. prausnitzii* and disease activity^18^,^56^,^57^. *F. prausnitzii* is known to promote intestinal health by producing butyrate, thus these results suggests an important role of this SCFA in disease activity^58^.

In line with other studies, *B. fragilis* was also found to be an important bacterial species to distinguish between patients in remission and patients with active disease^26^,^59^. Within a longitudinal pilot study, we previously showed a strong increase in the relative abundance of *B. fragilis* in two out of ten CD patients progressing from remission to an exacerbation^24^. Although *B. fragilis* is known as a commensal bacteria with anti-inflammatory properties, a recent study suggests that enterotoxigenic *B. fragilis* might play a role in active disease by increasing gut permeability^26^,^60^. Further studies need to be performed to investigate whether enterotoxigenic *B. fragilis* indeed is found more frequently in CD patients during exacerbation.

Papa *et al*. applied a RF-based algorithm to discriminate pediatric IBD patients in remission versus those with active disease and found Enterobacteriaceae (associated with disease activity) and Lachnospiraceae, *Ruminococcus, Roseburia* and *Blautia* (associated with remission) to be amongst the most important features to identify disease activity levels in pediatric IBD patients. In line with this study we found members of the *Lachnospiraceae, Ruminococcus, Roseburia* and *Blautia* amongst the most important OTUs, however we could not confirm whether these taxa were associated with remission exclusively. The discrepancies between our study and the study of Papa *et al*. could be due to a different population (children versus adults) as well as a different definition of disease activity (PCDAI and PUCAI versus a combination of clinical symptoms and CRP/FCP measurements). Moreover, Papa *et al*. collated CD and UC patients together to predict disease activity. Nonetheless, the performances of both models are similar, demonstrating the potential use of the microbiota as a predictive marker.

Since fecal calprotectin is known to correlate well with colonic inflammation, a correlation between fecal calprotectin and the 50 most discriminating OTUs was investigated. We found a very strong correlation between fecal calprotectin and the 50 most discriminating OTUs, which was however not significant. This might be due to a small number of samples. Fecal calprotectin has been reported to correlate well with colonic inflammation, but moderately with inflammation in the proximal colon and small bowel^9^,^10^. The current study however, thus clearly shows the potential of a bacterial profile consisting of a combination of OTUs as marker for disease activity. As perturbations of the intestinal microbiota are a potential pathophysiological factor in the development of exacerbations, it would be interesting to further investigate the potential of microbial profiling to monitor patients over time.

In conclusion, by applying random forest analysis we found that the fecal microbiota can be used to distinguish adult CD patients based on disease activity. A combination of 50 OTUs was found to be important in the discrimination between samples from remission and active disease, rather than specific bacterial taxa. Establishing a combination of key bacterial taxa unique to disease activity offers the opportunity to use simple and relatively inexpensive methods (*eg*. PCR-arrays) to assess disease activity. Furthermore, using the fecal microbiota as a disease activity marker can lead to new insights in the development of exacerbations and disease pathophysiology.

Further studies in which mucosal inflammation is assessed by endoscopy and prospective follow-up studies with IBD patients are warranted to validate our findings.

## Additional Information

**How to cite this article**: Tedjo, D. I. *et al*. The fecal microbiota as a biomarker for disease activity in Crohn’s disease. *Sci. Rep*. **6**, 35216; doi: 10.1038/srep35216 (2016).

## Supplementary Material

Supplementary Information

## Figures and Tables

**Table 1:** Baseline characteristics of CD patients (n = 71).

Number of samples per subject (%)	
Single sample	14 (19.7)
2 samples	21 (29.6)
3 samples	19 (26.8)
4 samples	8 (11.3)
5–8 samples	9 (12.7)
**Male** (%)	33 (46.5)
**Age** (in years; median, range)	44.0 (18–70)
**Disease localisation**^**1**^ (%)	
L1 (ileal)	23 (32.4)
L2 (colonic)	17 (23.9)
L3 (ileocolonic)	31 (43.7)
**Abdominal surgery** (%)	
(partial) colectomy	6 (8.5)
**Current smoking** (%)	14 (19.7)
**Age at diagnosis**^a^	
**A1 < 16y**	4 (5.6)
A2 17y-40y	47 (66.2)
A3 > 40y	20 (28.2)
**Disease phenotype**^**1**^	
B1 non-stricturing/nonpenetrating	52 (73.2)
B2 stricturing	10 (14.1)
B3 penetrating	9 (12.7)

^a^According to Montreal classification.

**Table 2:** Medication use, disease location and activity scores for active and remission samples^1^ (N = 194).

	Remission (n = 97)^a^	Active (n = 97)^a^
Medication use (%)^b^		
Mesalazine	14 (14.4)	9 (9.3)
Immunosuppressants	39 (40.2)	37 (38.1)
Biologicals	68 (70.1)	44 (45.4)
Antibiotics^b^	3 (3.1)	0 (0.0)
Disease location (%)^c^		
L1 (ileal)	46 (47.4)	23 (23.7)
L2 (colonic)	11 (11.3)	24 (24.7)
L3 (ileocolonic)	40 (41.2)	50 (51.5)
Fecal calprotectin^d^	14.0 (14.0–98.0)	582.0 (259.0–4900.0)
Serum CRP^d^	1.0 (0.0–4.7)	5.4 (0.9–175.0)
Clinical activity index (HBI)^d^	1.0 (0.0–4.0)	(0.0–15.0)

^a^194 samples were collected from 71 CD patients.

^b^Used between 1–3 months prior to sampling moment.

^c^According to Montreal classification.

^d^Continuous variables are expressed as median (range).
