# Supplementary material for: The fecal microbiota as a biomarker for disease activity in Crohn’s disease
Source: Sci Rep. 2016 Oct 13;6:35216. doi: 10.1038/srep35216 (PMC5062155; doi:10.1038/srep35216)
Supplement: Supplementary Information [file srep35216-s1.pdf]

The fecal microbiota as a biomarker for disease activity in Crohn's disease

Danyta.I.Tedjo[1,2], Agnieszka Smolinska[3], Paul H. Savelkoul,[2] Ad A. Masclee[1], Frederik J. van Schooten[3]

Marieke J. Pierik[1], John Penders[2]*, Daisy M. A. E. Jonkers[1]*

[1]School of Nutrition and Translational Research in Metabolism (NUTRIM), Division Gastroenterology-Hepatology, Maastricht University Medical Center+, Maastricht, The Netherlands.

[2]School of Nutrition and Translational Research in Metabolism (NUTRIM), Department of Medical Microbiology, Maastricht University Medical Center+, Maastricht, The Netherlands.

[3]School of Nutrition and Translational Research in Metabolism (NUTRIM), Department of Pharmacology & Toxicology, Maastricht University Medical Center+, Maastricht, The Netherlands.

- ***Shared last authorship**

Supplementary Figure 1A

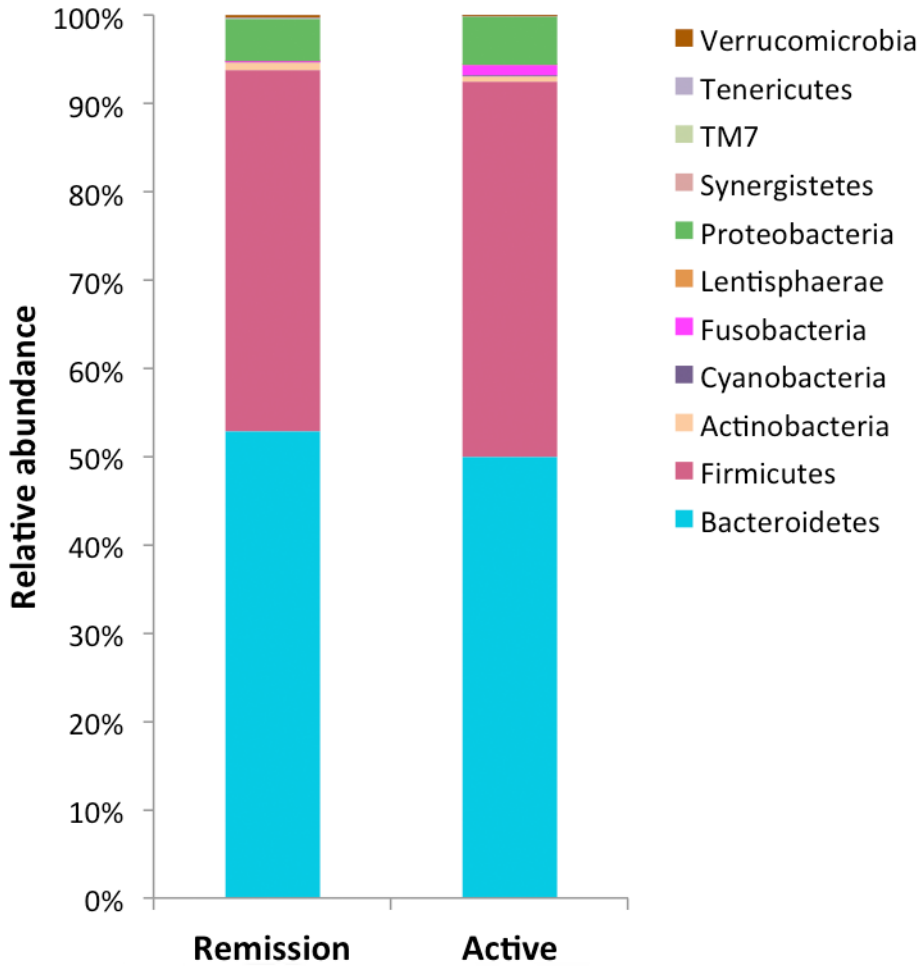

Supplementary Figure 1B

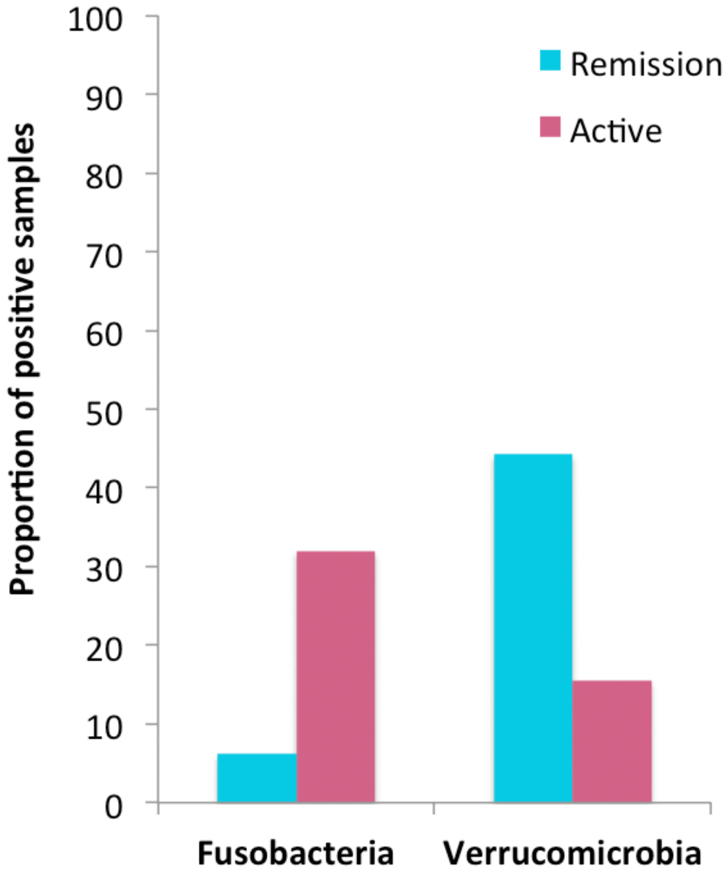

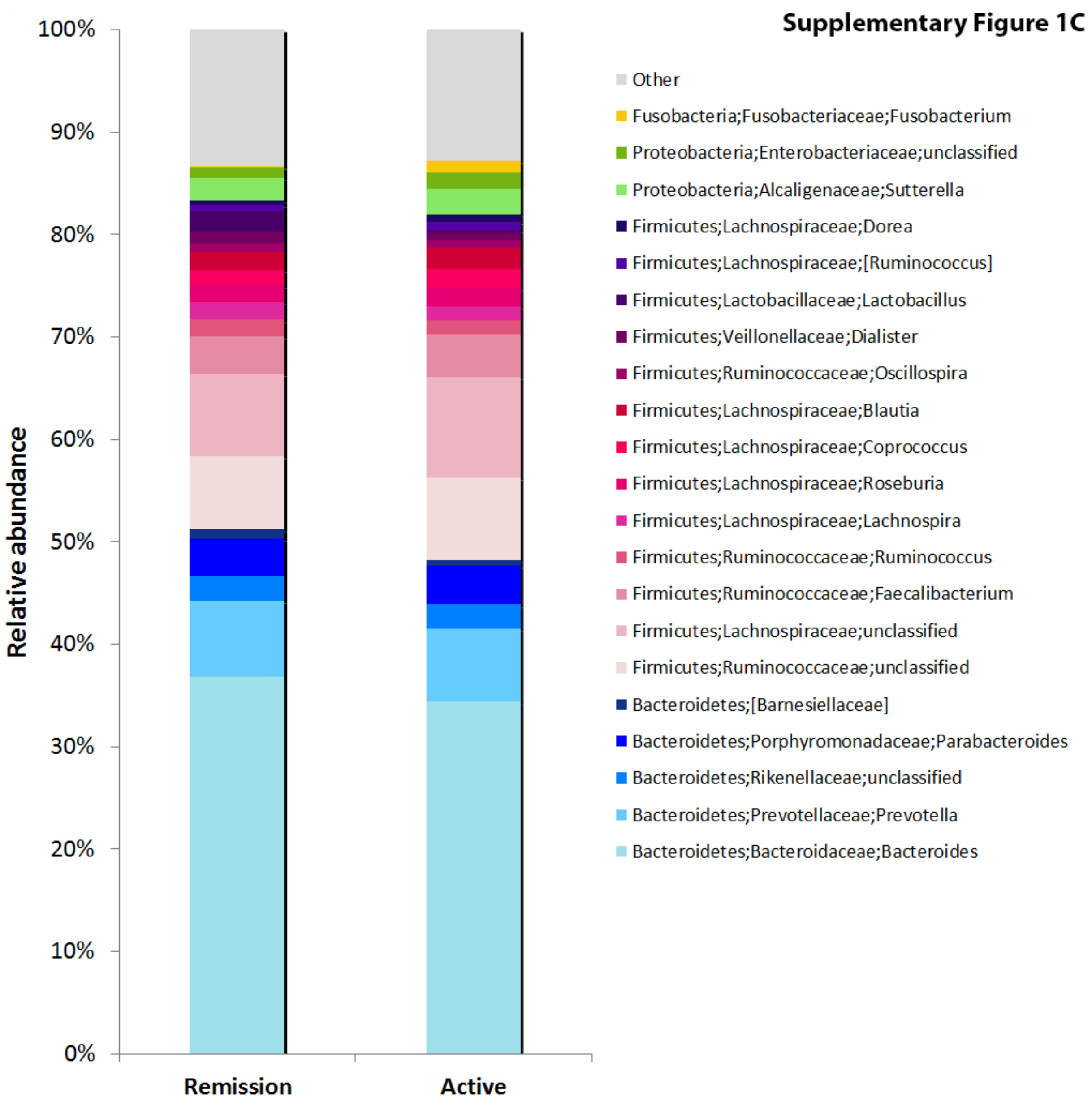

Supplementary Figure 1. Microbial composition of samples from Crohn's Disease patients during remission and active disease. A. Relative abundance of bacterial phyla. B. Proportion of samples containing Fusobacteria and Verrucomicrobia. C. Relative abundance of the most dominant bacterial genera (colored according to the respective phyla Bacteroidetes (blue), Firmicutes (pink - red – purple), Proteobacteria (green), Fusobacteria (yellow).

Supplementary table S1. Proportion of samples positive and mean (std) read numbers in positive samples for each of the discriminatory set of 50 OTUs for individuals in active and remission.

	Active CD samples (N=97)		Remission CD samples (N=97)	
**OTUs numbers**	**Number of samples positive for OTU**	**Mean read number (STD)***	**Number of samples positive for OTU**	**Mean read number (STD)***
2949328	54	11,06 (22,5341808854039)	30	12,40 (16,1001177978128)
3439402	30	15,17 (35,1990236280471)	22	13,86 (31,4904300724393)
192225	23	2,04 (1,63702226534437)	9	1,56 (0,52704627669473)
289449	31	4,81 (5,21164948193762)	9	2,11 (1,05409255338946)
4381555	22	5,64 (8,79147560367674)	8	11,38 (13,8763673714907)
292216	21	1,43 (0,870139561876632)	10	1,10 (0,316227766016838)
2447244	38	5,63 (9,09832492047101)	22	5,05 (5,35836926798828)
586453	5	1,40 (0,547722557505166)	22	1,36 (0,902137912878521)
193446	22	1,55 (0,962500351380158)	20	1,35 (0,812727700887249)
2771073	27	1,30 (0,541707756495413)	14	2,57 (2,40877516638598)
363348	24	1,71 (1,73152776639507)	10	1,10 (0,316227766016838)
4234212	13	2,31 (1,9315199276138)	38	7,97 (15,6265312194241)
215231	8	1,13 (0,353553390593274)	25	1,56 (0,820568908339411)
178920	19	1,26 (0,452413928358864)	27	1,41 (0,747264717757073)
583656	25	3,92 (3,49904748943671)	2	1,00 (0)
180610	25	1,28 (0,541602560309064)	12	1,17 (0,577350269189626)
330458	27	1,89 (2,00640001635791)	24	1,63 (1,17260393995586)
179055	23	2,13 (1,57550418556574)	11	1,27 (0,467099366496914)
4371302	27	1,52 (1,12216721537356)	22	1,86 (1,90976075653108)
177732	9	2,33 (1,5)	19	1,37 (0,683985568056769)
362997	6	6,33 (9,81155781039212)	23	4,48 (4,27327386967152)
4460021	6	1,83 (1,32916013582513)	33	2,55 (2,69363728407925)
183824	24	1,42 (0,775531608229038)	18	1,28 (0,460888598962477)
195093	32	1,63 (1,07012209131602)	14	1,50 (0,85485041426511)
184021	28	2,07 (1,84448269227889)	24	1,96 (1,68055805086252)
187782	21	1,33 (0,658280588604383)	10	2,00 (2)
182903	27	1,81 (1,07549763211973)	17	1,76 (1,25146972419961)
4407387	22	1,68 (0,994574023980865)	9	1,11 (0,333333333333333)
178465	19	1,95 (1,61498827689334)	24	2,46 (2,37704907792005)
4471854	43	1,77 (1,26936822122784)	40	1,73 (1,26059611458332)
107044	30	8,80 (20,0935742001748)	19	37,00 (56,6048682633492)
158331	11	2,91 (2,07145096270925)	32	3,84 (3,20392274684362)
191974	35	1,66 (1,34913604474378)	17	1,53 (0,874474632195206)
179107	11	1,27 (0,646669790682863)	19	1,21 (0,535303379031311)
113909	49	2,94 (3,38752714174432)	33	2,79 (2,63103562971855)
4390134	33	6,76 (10,5030659304507)	22	9,23 (19,6757811442485)
360995	23	1,30 (0,558795995356872)	16	1,06 (0,25)
192746	38	4,47 (5,17131820773871)	28	1,68 (1,46700933947825)
3797933	26	1,15 (0,464095480892257)	9	1,33 (0,707106781186548)
195029	27	2,93 (5,2909641838045)	12	2,33 (1,77525072919719)
189007	27	1,30 (0,541707756495413)	19	1,16 (0,374634324632678)
187894	28	2,43 (2,20149059651883)	12	1,17 (0,389249472080762)
190990	74	10,36 (15,5559743395495)	52	9,06 (9,27449266418271)
181056	32	3,38 (2,73271695121977)	35	2,94 (4,09406210722494)
191222	11	1,27 (0,646669790682863)	30	2,20 (1,93693679244601)
198521	27	1,96 (1,34397463006852)	22	1,59 (0,908116360757433)
366794	30	2,67 (3,42740812229615)	21	4,14 (5,91849401694143)
4456702	4	2,25 (2,5)	23	3,13 (4,71269415154516)
183873	21	1,33 (0,730296743340222)	7	1,57 (0,786795792469443)
2731539	22	3,36 (5,12273601139316)	7	46,43 (75,3720928524104)

*Mean (STD) read numbers are based upon samples positive for the specific OTU only.
